# Kinetic Barrier to Enzyme Inhibition Is Manipulated by Dynamical Local Interactions in *E. coli* DHFR

**DOI:** 10.1021/acs.jcim.3c00818

**Published:** 2023-07-26

**Authors:** Ebru Cetin, Tandac F. Guclu, Isik Kantarcioglu, Ilona K. Gaszek, Erdal Toprak, Ali Rana Atilgan, Burcu Dedeoglu, Canan Atilgan

**Affiliations:** † Faculty of Engineering and Natural Sciences, Sabanci University, Tuzla 34956, Istanbul, Turkey; ‡ Department of Pharmacology, University of Texas Southwestern Medical Center, Dallas 75390, Texas, United States; § Department of Chemistry, Gebze Technical University, Gebze 41400, Kocaeli, Turkey

## Abstract

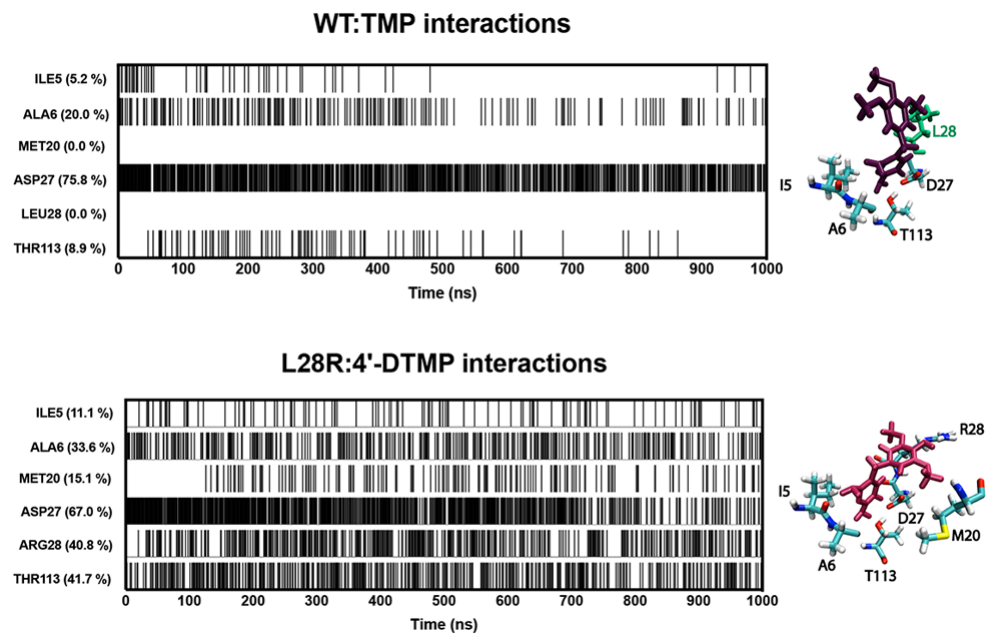

Dihydrofolate reductase (DHFR) is an important drug target and a highly studied model protein for understanding enzyme dynamics. DHFR’s crucial role in folate synthesis renders it an ideal candidate to understand protein function and protein evolution mechanisms. In this study, to understand how a newly proposed DHFR inhibitor, 4′-deoxy methyl trimethoprim (4′-DTMP), alters evolutionary trajectories, we studied interactions that lead to its superior performance over that of trimethoprim (TMP). To elucidate the inhibition mechanism of 4′-DTMP, we first confirmed, both computationally and experimentally, that the relative binding free energy cost for the mutation of TMP and 4′-DTMP is the same, pointing the origin of the characteristic differences to be kinetic rather than thermodynamic. We then employed an interaction-based analysis by focusing first on the active site and then on the whole enzyme. We confirmed that the polar modification in 4′-DTMP induces additional local interactions with the enzyme, particularly, the M20 loop. These changes are propagated to the whole enzyme as shifts in the hydrogen bond networks. To shed light on the allosteric interactions, we support our analysis with network-based community analysis and show that segmentation of the loop domain of inhibitor-bound DHFR must be avoided by a successful inhibitor.

## Introduction

Antibiotics play a critical role in the treatment and prevention of bacterial infections by inhibiting the growth of these organisms in the host. The discovery of antibiotics has been a breakthrough for mankind by reducing mortality and morbidity from infectious diseases.^1^ However, antibiotic resistant bacteria have emerged due to the often unnecessary and indiscriminate use of antibiotics; in fact, only a few years may suffice for a new drug to become ineffective in the clinic.^2,3^ New generation antibiotics are being developed to overcome the resistance problems against existing ones, but bacteria fight back by developing resistance against these new agents.^4^ Thus, it is of great importance to elucidate the mechanisms that cause resistance and accordingly design drugs that will impede the evolution of resistance.

**Figure 1:**
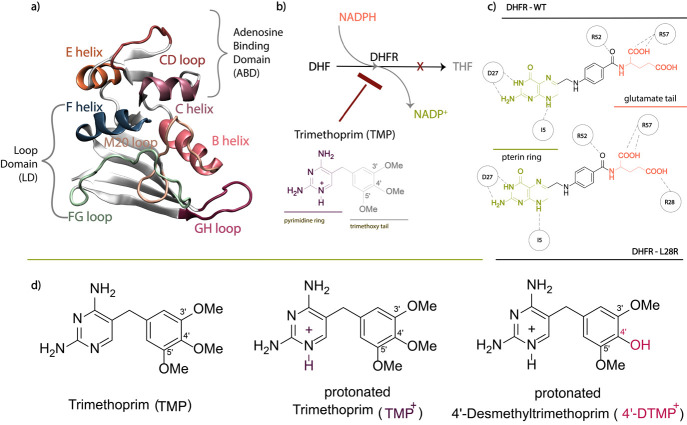
DHFR structure, mechanism, and its binding partners studied in this work. **a)** DHFR structure of the last snapshot of the TMP bound trajectory of the wild type structure, WT^TMP^. ABD spans residues 38–104, while LD comprises the rest. Loops are identified as M20 (9–24), CD (64–71), FG (116–132), and GH (142–150), whereas the helices are named B (24–36), C (43–51), E (79–86), and F (95–106). **b)** Schematics of the catalytic reaction of DHFR and its inhibition by TMP. **c)** Enzyme–substrate interactions. **d)** TMP and its derivative were studied in this work.

The design of new and more active drugs based on a knowledge of their mechanism of action plays a central role to overcome antibiotic resistance.^5,6^ Dihydrofolate reductase (DHFR) is an enzyme that has been frequently studied in this framework since it is widely found in all living organisms and has an important role in cell metabolism.^7,8^ In fact, due to its key biological function, it is an important therapeutic target for anticancer, antimalarial, and antibacterial medicine.^9−14^ DHFR is necessary for cell growth due to its role in the production of DNA precursors. It catalyzes the reduction of dihydrofolate (DHF) in the presence of the cofactor NADPH to tetrahydrofolate (THF), the precursors for purine and thymine. *Escherichia coli* DHFR (Figure 1a) has 159 residues and features eight β strands and four α helices, forming an α/β structure arranged into the adenosine binding (ABD) and loop (LD) domains.

Trimethoprim (TMP, Figure 1b) is a widely used antibiotic compound to treat various bacterial infections.^15,16^ TMP binds the orthosteric site of DHFR, inhibiting the reduction of DHF to THF (Figure 1b), an essential precursor in the synthesis of nucleic acids.^17^ The affinity of TMP for bacterial DHFR is much greater than that of the vertebrate enzyme, making this drug highly effective.^18^ On the other hand, its efficacy is limited due to the TMP resistance acquired by bacteria.^19^

Pathogenic bacteria can rapidly render antibiotics ineffective through the acquisition of mutations that lead to resistance. Understanding the evolutionary processes of these mutations is of great importance for the discovery of next-generation drugs. Toward this end, it was shown via evolutionary experiments, that it is possible to observe antibiotic resistance emerging in the DHFR encoding gene of bacterial cultures in the laboratory with a device called “morbidostat”.^20,21^ It was later shown that there was a disproportionate amount of resistant strains carrying the L28R mutation as the first mutation^22^ and has therefore been used as part of studies for understanding antibiotic resistance.^23^ Molecular dynamics (MD) simulations and biochemical experiments carried out in our group have shown the unique resistance mechanism of the L28R mutant.^22,24^ The extra hydrogen bonds formed dynamically between the L28R mutant (via residues R52, R57, and especially R28) and the glutamate tail of the DHF substrate lead to an increase in substrate affinity (Figure 1c), thus indirectly leading to TMP resistance. Based on this observation, we hypothesized that a more effective drug could be developed against strains carrying the L28R mutant by making a modification with a polar group at the para and meta positions of the trimethoxybenzene ring of TMP that would induce additional electrostatic and/or hydrogen-bonding interactions (Figure 1d).

Using this rationale, we synthesized an L28R-specific inhibitor by modifying the methyl group at the para (4′) position to a polar hydroxyl group.^25^ Among several other modifications, this new inhibitor (4′-deoxy trimethoprim; 4′-DTMP) was the only TMP derivative that was effective on both the wild type (WT) and the L28R mutant enzyme. Furthermore, under 4′-DTMP selection, the L28R mutation is largely eliminated, diverting the genetic trajectories to less resistant genotypes. In fact, the derivative exhibited 30–90-fold enhanced antimicrobial activity against isogenic *E. coli*-L28R compared to native TMP molecule. This study is important in terms of showing that it is possible to impede evolution of antibiotic resistance by blocking plausible evolutionary pathways with the most frequently observed mutant specific inhibitor (see Figure 4c in ref (25)). Therein, TMP-bound WT and L28R structures were also elucidated by X-ray crystallography where TMP is bound to the orthosteric site.^25^ In another study, we also showed that substrate-enzyme complex (DHF-bound DHFR) is synchronized at the precatalytic step and this behavior is governed by the active site residues which play a gating role, but the effects are propagated to the whole enzyme.^26^

If utilized to monitor the differences in the dynamics of a protein under varying conditions, MD simulations can inform on not only the thermodynamics but also kinetics of the system.^27−29^ Therefore, to inspect the mechanistic effects of the inhibitor derivatives, here, we study TMP and 4′-DTMP in the WT and L28R mutant protein environments. Since the binding event is controlled by the hydrogen bonds, we first analyze the orthosteric site interactions for the WT and L28R mutant enzymes for each inhibitor. We supplement these with free energy difference calculations using the free energy perturbation (FEP) method which are also supported by experimental measurements.^30,31^ We then expand our analysis protein-wide, by inspecting both the hydrogen bond occupancies and the formation of dynamical communities in the residue networks formed in this protein.^26,32^

## Materials and Methods

### Parametrization of 4′-DTMP

Trimethoprim derivatives were parametrized using the Force Field ToolKit (ffTK)^33^ plugin implemented in Visual Molecular Dynamics (VMD).^34^ The protocol used in our previous study^24^ to parametrize TMP and TMP^+^ was followed herein for the TMP derivative. All quantum mechanical calculations necessary for this protocol (optimization of charges, bonds, angles, and dihedrals) were performed by Gaussian09.^35^

Although it is known that TMP is neutral in water, it may also be in protonated form in the local protein environment depending on the mutant.^36^ In our previous study, the simulations were conducted in the presence of both neutral and protonated TMP (TMP^+^), and the protonation state was determined according to the maximum stabilization of the drug in the active site of DHFR.^24^ Similarly, both neutral and protonated states of 4′-DTMP structures were parametrized in this work, and the resulting parameters are given in Table S1. We find the protonation state of 4′-DTMP to be the same as TMP; *i.e*., it is protonated when bound both to the WT and to the mutant enzyme according to its hydrogen bonding profile (Figure S1). Therefore, in this study, only results from the protonated forms will be discussed.

### System Preparation

All simulations were conducted with the NAMD program.^37^ The water box was set to the dimensions of 62 × 68 × 60 Å with a padding of 10 Å TIP3P water layer in each direction. The salt concentration was set to isotonic conditions (0.15 M) by using K and Cl ions. The crystal structure was used in the closed conformation (PDB code 6XG5 and 6XG4) as the initial structure,^25^ and the L28R mutation was introduced using the VMD Mutator Plugin.^34^ Charmm36 parameter set^38^ for proteins was utilized. The cofactor NADPH was present in all simulations. The systems were minimized for 10 000 steps. Particle mesh Ewald sum was utilized to calculate long-range electrostatics with a cutoff distance of 12 Å and a switching distance of 10 Å. The RATTLE algorithm was applied, and the Verlet algorithm was used with a time step of 2 fs. The temperature was controlled by Langevin dynamics with a dampening coefficient of 5 ps^–1^. The pressure was set to 1 atm, and the pressure was regulated by the Langevin piston. The resulting structures were subjected to two 210 ns long production runs as duplicates and a 1 μs long run in the NPT ensemble. As in our previous work, the first 10 ns part of the trajectories was discarded for equilibration. The root mean squared deviation (RMSD) of the 210 ns-long and 1 μs-long simulations are shown in Figures S2 and S3, respectively. The root mean fluctuation (RMSF) values are reported as averages over all 200 ns long chunks of the trajectories (Figure S4). RMSD values in the equilibrated portion of the trajectories are 1.16 ± 0.19 and 1.28 ± 0.20 for protonated WT^TMP^ trajectories.

### Analysis of Hydrogen Bonds

The hydrogen bonds were initially obtained via the Hydrogen Bond plugin implemented in VMD by setting the criteria for the formation of hydrogen bonds with an acceptor–donor heavy atom distance of 3.2 Å and a bond angle of 20°. Hydrogen bonds between the ligand and the protein were extracted using an in-house script that shows neighboring hydrogen bonds of selected residues, and the corresponding distances were calculated using a Tcl script on the TK console of the Visual Molecular Dynamics (VMD) program. For protein wide hydrogen bond occupancies, the method for acquiring hydrogen bonds was used as in ref (26) with an occupancy difference criteria of %20; these were the hydrogens bonds whose occupancies change by more than three standard deviations of the mean (σ = 6.6, Figure S5) which provide the fingerprints of the hydrogen bonds. To select those with significant deviations from the WT, we also calculated errors on these occupancy changes by splitting the 1 μs trajectory into five chunks of 200 ns each. Bonds that show less than 2σ fluctuation were chosen for further refinement (Table S2).

### Alchemical Free Energy Perturbation Calculations

Zwanzig’s formulation^30^ was followed for all the free energy difference calculations. A soft-core potential was introduced to the Lennard-Jones potential by truncating the potential function shifting van der Waals radius 2 Å for reducing singularities occurring during the calculations. Dual topology was used for the TMP^+^ to 4′-DTMP^+^ conversions. Initial conformations from seven different time points of the MD simulations were used in sampling to enhance the coverage of the potentially available conformational states. All calculations were carried out with the Charmm36 force field. Particle mesh Ewald sum was employed to control the electrostatics. Langevin piston was used for pressure control. Switching was implemented with a cutoff of 13 Å and a switching distance of 10 Å. The time step was set to 2 fs. The SHAKE algorithm was used for all bonds. Systems were equilibrated for 0.5 ns posterior to 500 steps of minimization. The window size was set to 0.2 ns, 0.1 ns being the window for equilibration. The systems were run for 32 windows in forward and backward simulations. Acquired results were merged and analyzed by the multistate Bennett-acceptance ratio (MBAR)^31^ method using the established protocol.^39^

### Dynamical Community Composition

Residue networks (RNs) were built by using a frame for every 10 ns of the last 100 ns part of each trajectory. C_β_ (C_α_ for Glycine) atoms were taken as nodes for the amino acids. C2 atom (Table S1) was used as a node for TMP and 4′-DTMP. Hence, the graphs were constructed with a total number of 160 nodes, and 6.7 cutoff was used to add a link between a pair of nodes. Resulting RNs were undirected and did not have self-loops. Girvan-Newman algorithm^40,41^ was employed to separate the protein into six communities (Ω = 6) in each snapshot. This algorithm searches for communities by breaking the most central (highest betweenness centrality) edge in each iteration. The calculations were repeated for the equally spaced frames obtained from the MD trajectories. Three well-separated nodes were then selected to visualize the dynamical communities. These represent two locations at the outskirts of each of the LD (N142) and ABD (S64), and one bridging the two domains (I94). Red-green-blue (RGB) scores were accumulated on these three residues such that for each snapshot, if a residue was in the same community as one of these three residues, the color belonging to that residue (I94, N142, and S64 for red, blue, and green, respectively) was incremented by one. Dynamic community composition was then projected on the three-dimensional protein structure by using the normalized (to 256) color score of each residue. For example, if a residue was always in the same community as I94 (RGB value 256,0,0), it was colored red; if another residue was in the same community with I94 and N142 50% of the time each (RGB value 128,0,128), it was colored magenta. See ref (32) for more details.

### Protein Overexpression and Purification

The L28R mutation in folA gene was constructed by using the Quick-Change Site-Directed Mutagenesis kit (Stratagene). Six×HisTag was added at the C-termini of the protein sequence for the WT and L28R constructs. The constructs were cloned into expression plasmids pET24a-KanR. BL21 *E. coli* cells were transformed with pET24a-folA–6×HisTag and were grown overnight in selective media (LB + Kan) and then diluted 100 times into Terrific Broth (TB) media for further growth at 37 °C. Protein overexpression was induced when OD600 reached 0.6–0.8, using 250 mM isopropyl β-d-1-thiogalactopyranoside (IPTG) per 1 L of the medium, and the temperature was decreased to 18 °C for overnight growth, with 230 rpm shaking. Recombinant protein was purified using Ni-NTA columns (Qiagen), dialyzed overnight against 50 mM Tris-HCl, 300 mM NaCl, 0.5 mM tris(2-carboxyethyl)phosphine (TCEP), pH 8, and further purified using size-exclusion chromatography.

### Steady-State Kinetic Measurements

We followed the protocol outlined in our previous work.^22^ Briefly, in the DHFR reaction, reactants DHF (Sigma-Aldrich D7006) and NADPH (Sigma-Aldrich N7505) have light absorption at 340 nm wavelength, where the products THF and NADP^+^ do not absorb light at this wavelength. The reaction concentrations were quantitated using extinction coefficients of 28 mM^–1^ cm^–1^ for DHF at 282 nm and 6.22 mM^–1^ cm^–1^ for NADPH at 340 nm. The reactions were run in MTEN buffer (50 mM 2-(*N*-morpholino) ethanesulfonic acid, 25 mM tris base, 25 mM ethanolamine, 100 mM NaCl) pH 7.00, and 5 mM dithiothreitol (DTT, Fisher Scientific BP172-25) was added freshly before starting the reaction. MTEN solution with 150 nM DHFR protein and 200 mM NADPH was prepared. Reaction progression was measured using UV/vis Spectrophotometer (PerkinElmer LAMBDA 650) with 1 s time intervals for two cells. The first cuvette contained the reaction components (DHFR, DHF, and NADPH), and the second cuvette was the reference used for background correction which contained only MTEN and DTT. The reaction concentration for DHF in the first cuvette was 12.5 μM, and the data was collected until DHF was consumed completely, which happens when the curve reaches plateau. To observe the effect of TMP and 4′-DTMP on the reactions, 12.5 μM DHF consumption measurements were used as control. The consumption of 12.5 μM DHF was measured in either WT and L28R mutant in the presence of either TMP or 4′-DTMP. The inhibitor concentration was 500 nM in each case, and all measurements were done in duplicate.

## Results and Discussion

### Relative Binding Energies Reveal Similar Energetic Cost for 4′-DTMP and TMP Binding to WT and L28R Mutant

4′-DTMP was designed^25^ based on our observation that the L28R mutant of DHFR establishes dynamical hydrogen bonds with the substrate DHF in the precatalytic step^22,24^ and that a TMP derivative exploiting similar dynamics might be effective on both the WT and the mutant enzyme. This observed effect was shown to be of kinetic origin,^24^ as the free energy difference calculated for binding to WT vs L28R mutants was 1.2 ± 1.4 kcal mol^–1^ for DHF and 0.6 ± 1.1 kcal mol^–1^ for TMP. Therein, the calculations were carried out via steered MD simulations. Due to the similarity in the structures of TMP and 4′-DTMP, here we can calculate the thermodynamics of their relative binding with high accuracy using FEP.

**Figure 2:**
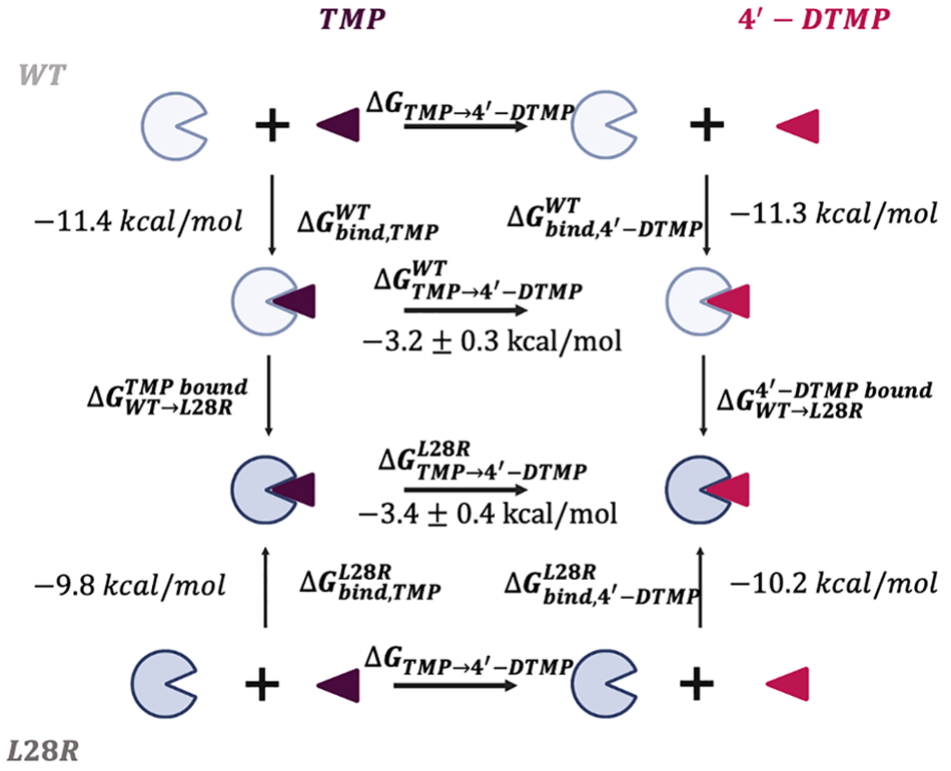
Illustration of mutation and ligand binding thermodynamic cycles of two inhibitors to DHFR. The upper and lower cycles represent the binding of ligands TMP and 4′-DTMP to WT and L28R, respectively. Enzymes are depicted in pacman style, representing WT in light gray and L28R in darker gray. The ligands of each type are represented by burgundy and fuchsia. Throughout the text, we kept the same coloring for TMP (burgundy) and 4′-DTMP (fuchsia). Vertical values are from experiments; horizontal ones are computed. Created with BioRender.com.

The thermodynamic cycle constructed to compute relative free-energy changes due to ligand binding is illustrated in Figure 2. In these cycles, the vertical energy differences (Δ*G*_bind, TMP/4′-DTMP_^WT/L28R^) can be obtained from experimental binding affinities using the relation Δ*G* = −*RT* ln *K*_*i*_ (Table 1). The horizontal ones (Δ*G*_TMP→4′-DTMP_^WT/L28R^) are the theoretical values obtained through FEP simulations.

**Table 1:** Experimental Binding Affinities and Corresponding Calculated Binding Free Energies

**protein**	**inhibitor**	***K***_***i***_**(nM)**^a^	**Δ*****G*** (kcal/mol)	**label**
WT	TMP	4.2 ± 0.5	–11.5 ± 0.1	Δ*G*_bind, TMP_^WT^
WT	4′-DTMP	5.1 ± 1.0	–11.4 ± 0.1	Δ*G*_bind, 4′-DTMP_^WT^
L28R	TMP	65.0 ± 7.5	–9.9 ± 0.1	Δ*G*_bind,TMP_^L28R^
L28R	4′-DTMP	34.3 ± 2.9	–10.3 ± 0.1	Δ*G*_bind, 4′-DTMP_^L28R^

a From ref (25).

Experimentally, we find that, while binding to the protein is tighter in the WT than the mutant, they are all in the nanomolar range for both inhibitors. Moreover, our calculations have led to the results, Δ*G*_TMP→4'-DTMP_^WT^*=* – 3.2 ± 0.3 kcal/mol and Δ*G*_TMP→4'-DTMP_^L28R^ = −3.4 ± 0.4 kcal/mol.

The relative effect of the inhibitors may be obtained from the difference: ΔΔ*G* = Δ*G*_WT→L28R_^TMP bound^ – Δ*G*_WT→L28R_^4'-DTMP bound^. Employing the outermost and the innermost cycles from Figure 2, and plugging in the measured/calculated values we get,
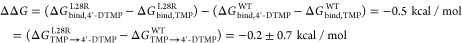
Thus, both the experiments of ref (25) and our calculations point out that the effect of the mutation is thermodynamically similar for the two inhibitors. Therefore, the 30–90-fold enhanced activity in the inhibition of the L28R mutant by 4′-DTMP we previously observed^25^ must have kinetic origins.

### Steady-State Kinetic Measurements Point out an Inhibitory Difference for TMP and 4′-DTMP

**Figure 3:**
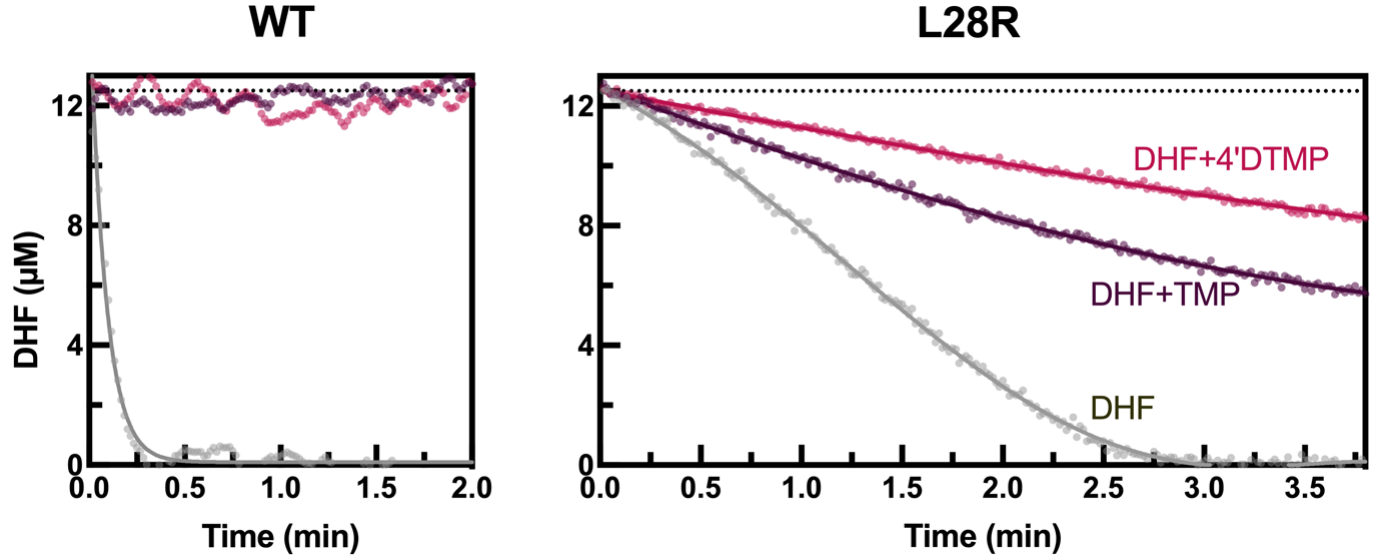
Kinetic assays for WT and L28R mutant enzyme. Initial DHF concentration in each case is 12.5 μM (dotted lines). Gray curves are the controls in the absence of inhibitors; colored curves are for the systems under competitive inhibition of the 500 nM drug. Experiments are done in duplicate; raw data are shown in circles; lines guide the eye.

In kinetic assays, we measured the consumption of DHF in the absence and presence of the inhibitors (Figure 3). The initial DHF concentration was 12.5 μM, and these assays were performed for WT and L28R mutant enzymes. DHF was consumed in 30 s in control experiments in the absence of any inhibitor for WT DHFR. When 500 nM TMP and 4′-DTMP molecules were added, we did not observe any DHF consumption; *i.e*., the enzyme is completely inhibited. This is in accordance with the 4–5 nM *K*_*i*_ values of these drugs for the WT. On the other hand, in the DHF consumption control experiment for the L28R mutant, we observed a slower trend continuing for a few minutes. Since WT protein is optimized for the environmental conditions, the slower reaction kinetics for L28R is expected; in fact, the more resistant mutants of bacteria are likely to grow slower than WT, introducing a resistance cost.^42^ When 500 nM inhibitors are added to the mutants, both are able to introduce a slowing down in product release, more so for 4′-DTMP than for TMP. Thus, the slight edge introduced by 4′-DTMP on the L28R mutant finds its origins in these kinetic differences. We next explore the modified interactions in the dynamics of inhibitor bound DHFR to illuminate this point.

### Hydrogen Bond Occupancies Differentiate Binding Modes of the Two Inhibitors

To elucidate the mechanism of binding of the new inhibitor and thus to highlight the interactions responsible for its enzymatic activity, a detailed hydrogen bond analysis in the WT and L28R mutant of *E*. *coli* DHFR with TMP derivatives has been conducted by analyzing the MD simulations for each system.

**Figure 4:**
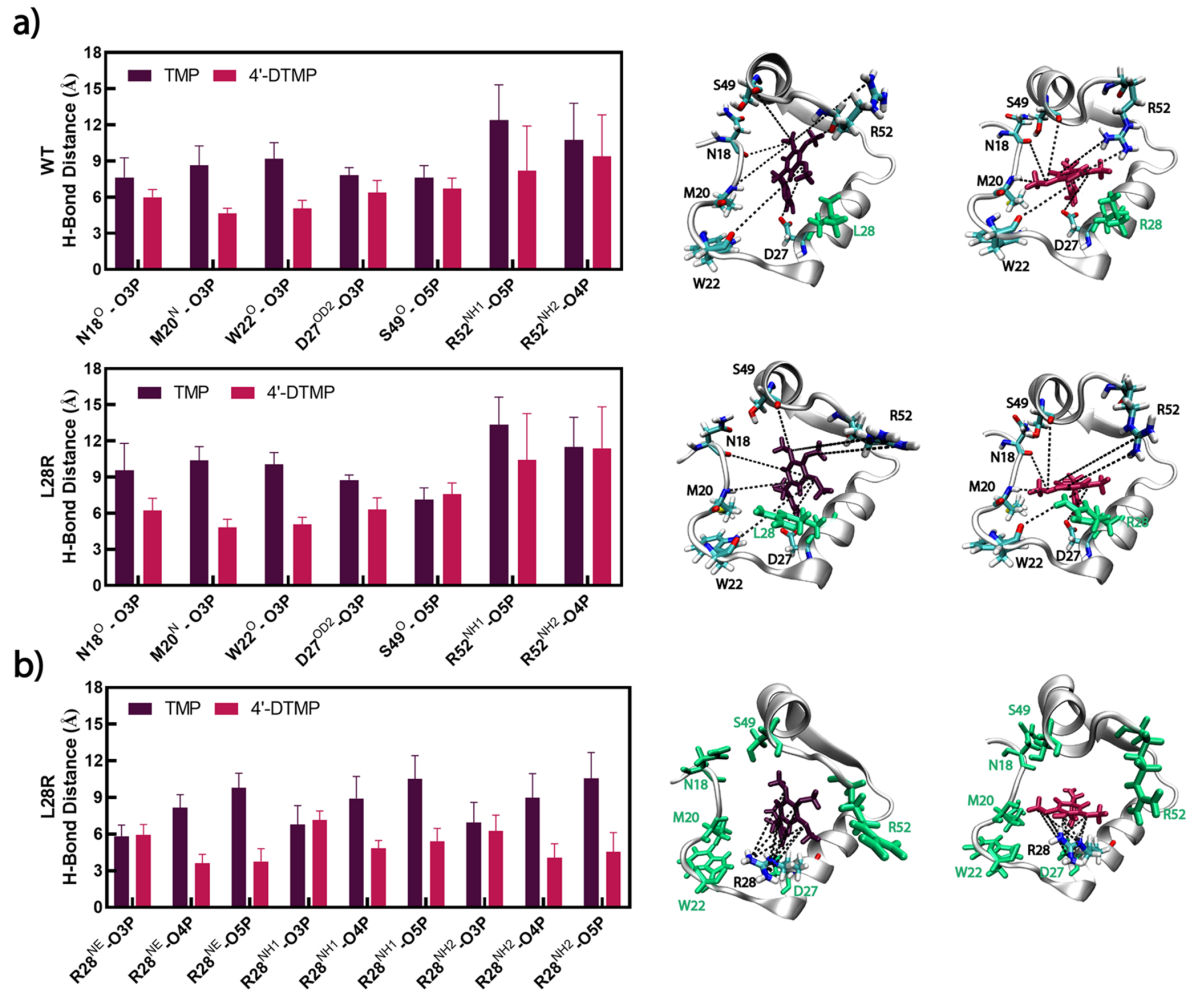
L28R variant of DHFR forms additional and stronger hydrogen bonds with 4′-DTMP compared to TMP. Average distances between the ligands and the binding site of WT and L28R for selected atom pairs; since the hydrogen bond distance definition is 3.2 Å, values less than 6 Å imply formation of frequent hydrogen bonds in the dynamical trajectories. (a) Trimethoxybenzene tail and the surrounding loop interactions; (b) R28 interactions with the ligand. Zoomed-in representations of these interactions are also displayed on the structures on the right, with the same color code for the inhibitors.

In the crystal structure of WT^TMP^ and L28R^TMP^ (PDB codes 6XG5 and 6XG4(25)), the pyrimidine ring of TMP is stabilized by the backbone oxygen atoms of I5 and I94 and the side chains of D27, Y100, and T113, whereas the trimethoxybenzene ring is stabilized via the side chain of S49 (also see Figure S1). We monitored the average hydrogen bond distances in the WT and the L28R mutant in the presence of TMP and its derivative 4′-DTMP. The hydrogen bonds established between the 2,4-diamino pyrimidine ring of the ligand and the active site residues of the protein are highly conserved, contributing to the stabilization of the ligand within the active site of DHFR (Figure S1). While the pyrimidine ring of the ligand is stabilized with strong hydrogen bonds both in WT and in L28R, we observe a dynamic stabilization of the trimethoxybenzene ring of the ligand. Residues N18, M20, W22, D27, S49, and R52 come in proximity occasionally and sometimes lose their contact resulting in larger average values for the contacts determining hydrogen bond distances. In particular, N18, M20, and W22 show significant changes in hydrogen bonding distances in both WT and L28R for 4′-DTMP (Figure 4a). On the other hand, for the L28R mutant, the most fundamental difference is the interactions of oxygen atoms at the 4′ and 5′ positions on the inhibitors with the R28 atoms whereby frequent strong interactions are formed with 4′-DTMP, which do not form with TMP (Figure 4b).

**Figure 5:**
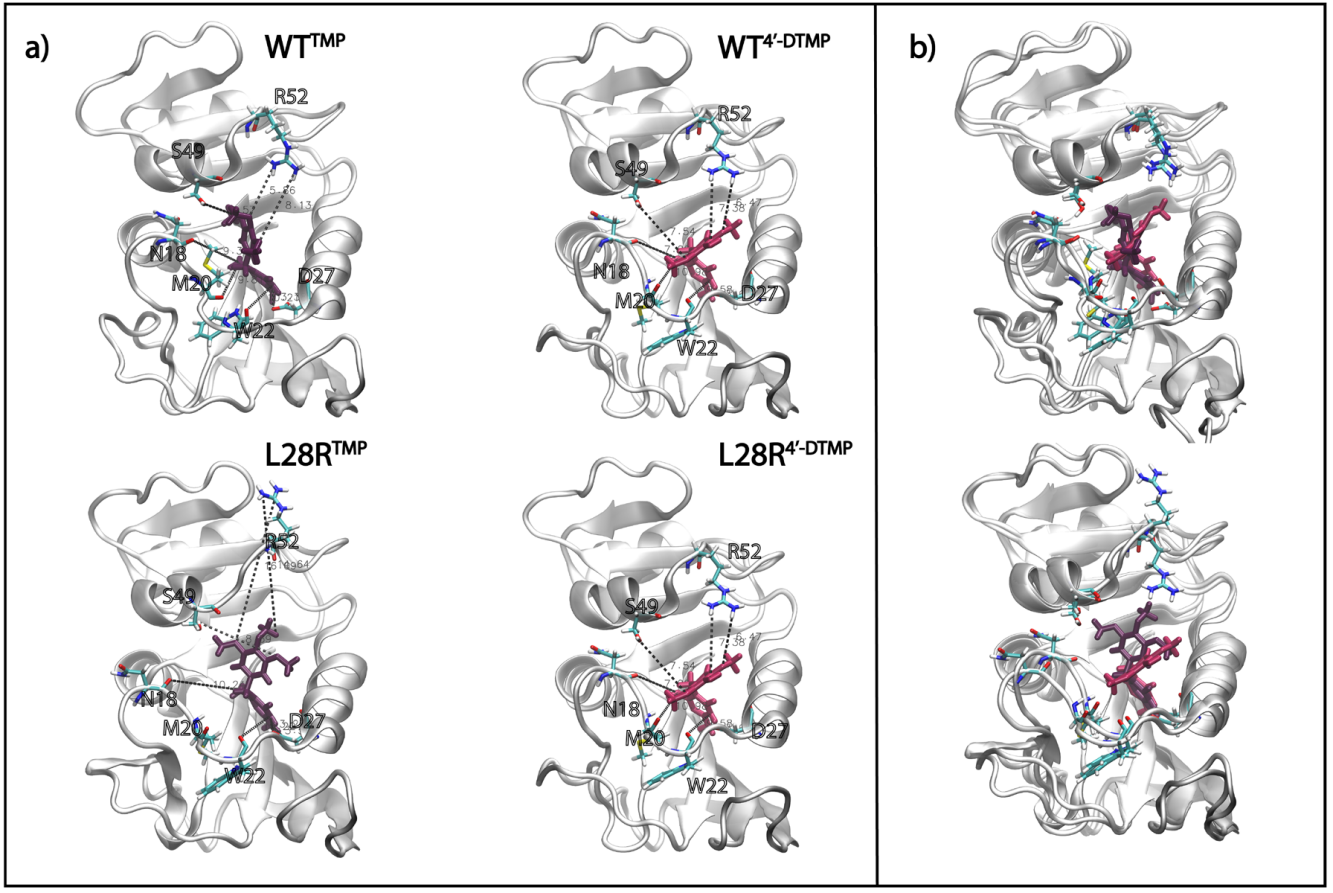
Inhibitors share enzyme interactions but prefer different dynamic conformations. (a) Interactions of active site residues with TMP (purple) and 4′-DTMP (pink) in both WT and L28R at the end of one of the replicated 210 ns trajectories. Note how the R52 side chain that turns away from the binding cavity in the L28R^TMP^ system is held in place by the 4′-DTMP. Also, TMP has alternative conformations, while 4′-DTMP has a single stable pose. (b) Superposition of the interactions of the inhibitors with the WT (top) and L28R variant (bottom).

That the pyrimidine ring in both WT and L28R shares the same binding partners while the trimethoxybenzene ring is stabilized by dynamic interactions in the latter is depicted in Figure 5. We observe a highly dynamic trimethoxy tail for TMP, even displaying rotational jumps between two ring conformations during a given 200 ns-long trajectory, constantly making and breaking hydrogen bond contacts. Conversely, 4′-DTMP is lodged in place due to the effect of the N18, M20, W22 and, in the case of L28R, R28 interactions.

**Figure 6:**
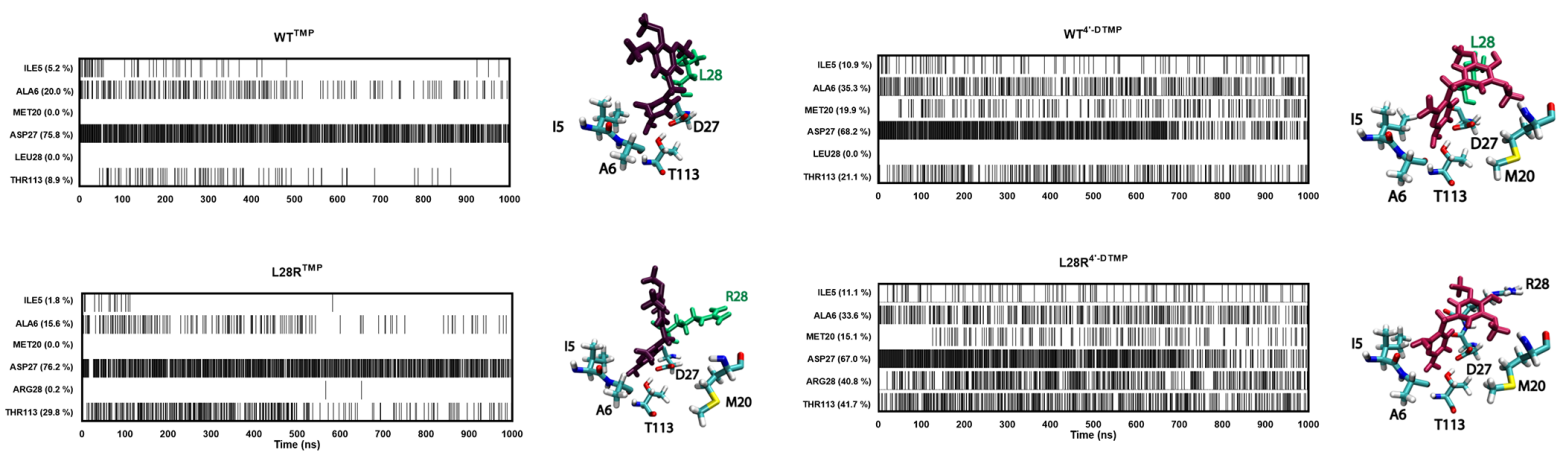
Enhancement of pyrimidine ring interactions through trimethoxy tail stabilization. Barcode graphs for the hydrogen bonds between binding cavity residues and TMP and 4′-DTMP for WT and L28R were obtained from the 1 μs-long MD trajectory; similar statistics hold for the duplicated 210 ns-long trajectories. A value of one is assigned if there is at least one hydrogen bond between any atom of the residue and the ligand and zero if there is no interaction. These interactions with the inhibitors are displayed on the structures on the right of each barcode graph.

Figure 6 displays the time trace of residues that are hydrogen bonded to the inhibitors for more than 10% of the time in any one of the trajectories. In all systems, the pyrimidine ring is stabilized predominantly by D27 supported by the dynamical interplay with I5, A6, and T113 residues. We observe that interactions are slightly decreased for D27 in 4′-DTMP proteins due to inclusion of additional binding partners. In the L28R mutant, there are additional interactions of the tail of 4′-DTMP due to positively charged arginine residue in the vicinity. With the stabilization of the tail of the ligand for 4′-DTMP, the pyrimidine ring is further immobilized in the active site, quantified with an increase in the occupancies, particularly for A6 (from 16 to 20% to 33–35%). In the presence of 4′-DTMP, there is a significant enhancement of the T113–inhibitor interactions, from 9 to 21% in the WT and from 30 to 42% in L28R. T113 engages the pyrimidine ring (Figure S1), and additional stabilization of the trimethoxy tail ensures further stabilization of this portion of the inhibitor.

### Changes in Local Dynamics Propagates to Total Enzyme Motions and Expose the Dynamical Mechanism of Action

We next question if these increased local dynamical interactions between the enzyme and the inhibitor permeate the rest of the protein by examining the cross-correlation maps (Figure S6). We find that for either of the inhibitor bound forms, the L28R mutation reinforces both positive and negative correlations observed across the enzyme (comparing upper and lower maps in Figure S6). On the other hand, the modification of the inhibitor from TMP to 4′-DTMP substantially increases the negative correlations of the GH loop (residues 142–150) with the rest of the protein (blue vertical/horizontal stripe in the second column correlation maps). In fact, 4′-DTMP suppresses correlations occurring elsewhere in the system (compare the shades of red/blue from left to right), except for the positive correlations between CD (residues 64–71) and GH loops. We note that we have previously observed that CD loop has allosteric effects on DHFR dynamics.^26^ Thus, although binding free energy differences are comparable, there are important mechanism changes due to both the mutation and ligand modification that are only reflected in the dynamics of the enzyme.

### Dynamic Interplay between Allosteric Regions Determines Enzyme Inhibition Outcomes

**Figure 7:**
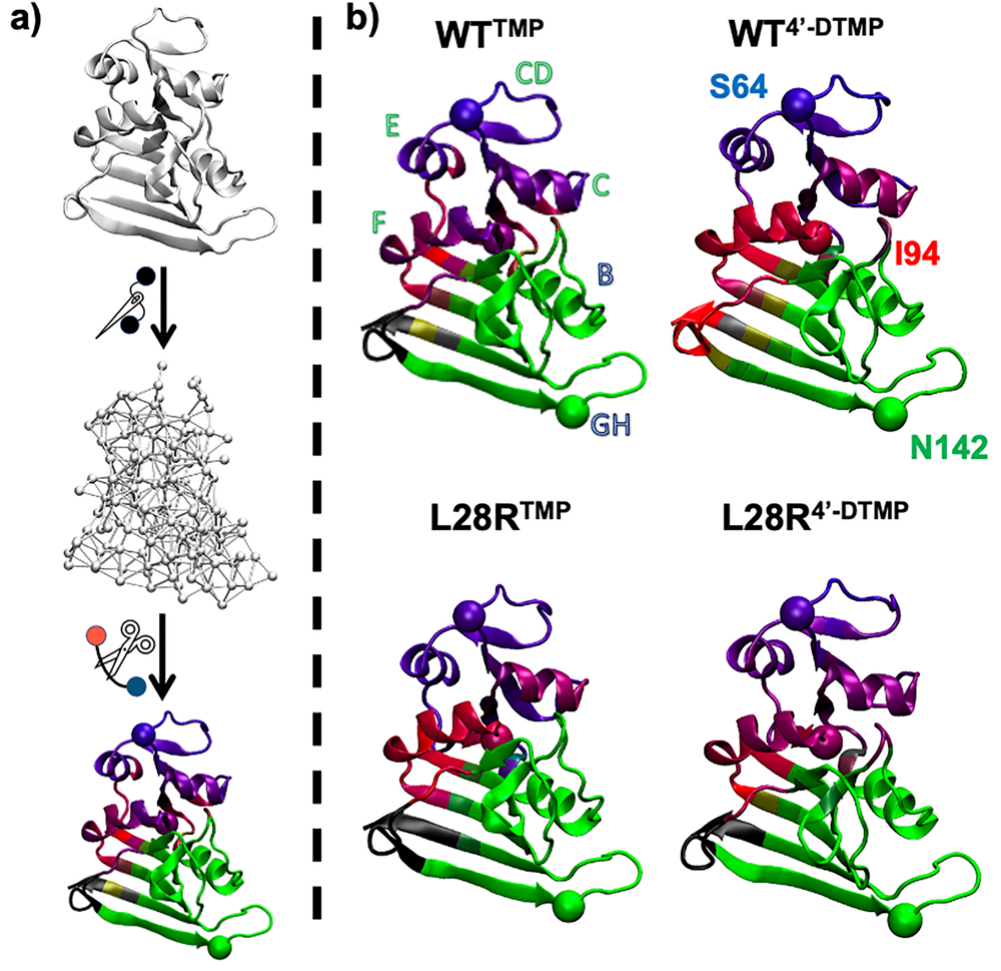
Distinct communities imply segmentation. a) Schematic summarizing community analysis. First, a protein structure is projected onto a graph by taking C_β_ (C_α_ for glycine) as nodes and connected by links if they are within a cutoff distance of 6.7 Å. Then, by iteratively cutting the most central links, segmentation into communities is computed for each MD snapshot. Coloring is directly proportional to a residue belonging to the selected reference RGB nodes over the entirety of the trajectory (I94 = red, N142 = green, S64 = blue). b) Dynamic communities illustrate the community sharing for WT and L28R bound forms with TMP and 4′-DTMP.

Our previous work on DHFR mutational landscape has irrevocably put forth that one needs to analyze the dynamical behavior of this enzyme in the presence of its various ligands to fully explain its behavior.^22^ To answer the question of how the different regions in the enzyme are connected, we first resort to construct a visual of the community structures in the trajectories. To achieve this, we have selected three residues that predominantly remain in separate communities and are also far apart from each other; S64, I94, and N142. These are assigned the color codes blue, red, and green, respectively. Residues that remain in the same community are represented as single colors (red, green, or blue); otherwise, they appear in mixed colors reflected on the three-dimensional protein structures in Figure 7.

We first note that in all the systems, the ABD is colored purple-magenta indicating that it shares communities with the binding site (red). Community structures indicate in L28R^TMP^ that the LD is split into three independent regions (pure red, pure green, and black colors) throughout the trajectory. This suggests that these regions are dynamically independent from each other, not sharing communication pathways during the time scale of the observations (1 μs). Thus, in L28R^TMP^ the binding site is isolated from the rest of the LD so that the allosteric role of the CD loop is abolished. This situation contrasts with the rest of the systems, where various other color tones emerge in the LD.

One can be conjectured that it is easier to separate the ABD from the LD in this scenario so that the barrier to inhibitor release is lowered, rendering the inhibition of L28R by TMP unsuccessful so that the protein remains functional. Based on this finding, we hypothesize that isolation of the communities belonging to the CD loop and E helix is necessary to confer resistance. Considering that TMP disrupts the function of WT DHFR, the purple-red color of F helix in WT^TMP^ and L28R^4'-DTMP^ further indicates that the community sharing between ABD and LD is another successful indicator of inhibition of the enzyme. Furthermore, the C-termini of WT^TMP^, L28R^TMP^, and L28R^4'-DTMP^ complexes do not share communities with other parts of the protein by being colored in black. This community structure might be one explanation as to why the C-terminus is independent of the rest of protein dynamics during the product release step.^43^ WT^4'-DTMP^ differs from the other three systems in this respect, where the C-terminus shares a community with I94 on the F helix.

**Figure 8:**
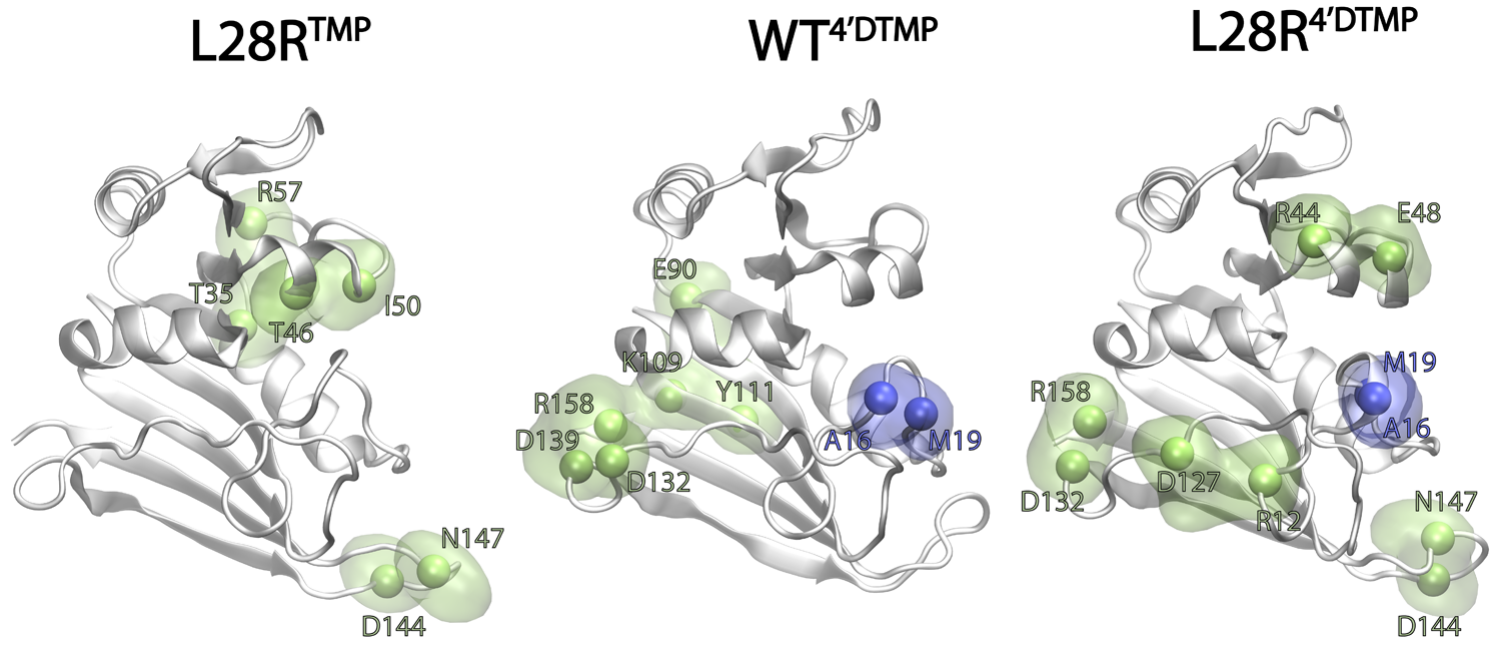
Hairpin formed on the M20 loop manipulates internal communication pathways of DHFR. Hydrogen bonded pairs whose occupancies change by more than 20% with respect to the WT^TMP^. Green: decrease in occupancy; blue: increase in occupancy.

We find that the dominant factors that contribute to the shifts in communities are those hydrogen bonds in the overall system that display significant changes in their occupancies. We thus resort to tracking residue pairs which consistently shift their hydrogen bond occupancies with respect to the WT^TMP^ by more than 20% (see Table S2 for selection criteria and process). The results are listed in Figure 8. In L28R^TMP^, the decreased occurrence (green spheres) of T46-I50 and T35-R57 in the ABD leads to the increased fluctuations in the C helix (Figure S4). The significant loss of the D144-N147 interaction in the GH loop of the LD further contributes to the long-range communication with the CD loop which modulates its allosteric character. Together, they lead to the isolated communities of the LD in Figure 7.

For the WT^4'-DTMP^ and L28R^4'-DTMP^ systems, there is generally a decrease in some of the C-termini interactions compensated by the newly formed A16-M19 interaction (Figure 8). The hairpin formed between A16 and M19 creates a hybrid conformation of the loop which is connected to the bound ligand. This rigidification of the M20 loop might be responsible for slowing down the inhibitor release step. While there is significant overlap in the residues involved at C-termini hydrogen bond occupancy loss, others require further inspection. The loss of the E90–K109–Y111 network in WT^4'-DTMP^ could be providing the flexibility necessary for increasing the communication pathways present in the enzyme, so that the allosteric communication between the CD–GH loops prevails. Otherwise, the systems need to acquire additional flexibility in the GH loop (see the bond D144–N147 in L28R^4'-DTMP^). With the introduction of R28 in L28R^4'-DTMP^, the further branching of binding partners introduces attenuation in the binding occupancies overall shifting M20 loop toward the binding site which results in the loss of R12-D127 bond. These motions then lead to the blending of the binding region motions with the ABD completely, reaching all the way to the CD loop, which we have previously predicted to be an important allosteric communicator in DHFR,^26^ also corroborated with enhanced cross correlations (Figure S6).

In sum, maintaining the delicate balance of the interaction network in DHFR seems to be the key to the success of an inhibitor and is a mechanism also exploited by resistance-conferring mutations. Thompson et al. find that the destabilization of the enzyme is found in most of the advantageous mutations and the mutants are thought to function through accelerated breathing motions which eases the way of product release.^44^ In fact, the increased hydrogen bond occupancy of 4′-DTMP with N18, M20, and W22 in the M20 loop is the only logical source of the hydrogen bond rupturing. Sawaya and Kraut point out that when the M20 loop changes conformation, the hydrogen bonds between the M20 loop and FG loop (G15-D122 in the closed state, E17-D122 in the open state) rupture and the new bond between GH and M20 loop (N23–S148 in the occluded state) forms.^45^ Here we see similar behavior between the domains, albeit not permanently as in those discussed X-ray structures but dynamically.

## Conclusions

DHFR is a highly flexible and dynamic protein with many allosteric residues.^46−48^ The effective TMP derivative, 4′-DTMP, developed specifically toward steering evolutionary trajectories away from the L28R mutation which emerges frequently under TMP pressure, is studied along with MD and FEP simulations. Using both experimental and computational means, we find that the two inhibitors are thermodynamically similar to comparable binding energy differences between the WT and L28R systems. This leaves alteration of the kinetics as the most plausible means for explaining the 30–90-fold enhanced antimicrobial activity^25^ which might be due to, e.g., modified dissociation pathways or increased kinetic barriers in the reaction steps. We therefore seek to understand the underlying dynamic causes of the effectiveness of the derivative. We first analyze the local interactions and find that the original design parameter that targeted a polar modification in TMP indeed caused the formation of extra hydrogen bonds with L28R, and these interactions were demonstrated in MD simulations.

For the inhibition of DHFR in the orthosteric site, we elucidated that dynamic stabilization of the inhibitor is crucial to slow down the functioning of the L28R mutant. Enhanced M20 loop interactions with the trimethoxy tail are triggered in addition to those intended with the R52 region during the design of the inhibitor (Figures 4a and 5). In addition, the enhancement of hydrogen bonds with the pyrimidine ring through A6 and T113 shows that there is also an intrinsic effect due to the charge density of the polar group added to the molecule. However, this effect is not significant, since we do not see any change in the inhibitory behavior in the WT.

We next focused on how these local interactions propagated to the total enzyme. Our community analyses showed that the community separation in L28R^TMP^ indicates a distinct communication regime utilizing segmentation of the enzyme, which causes the recovery of the function. On the other hand, the community composition of WT^TMP^ and L28R^4'DTMP^ displays long-range slow-motion communication, which results in inhibition. This is because, the further the coverage of the dynamical hydrogen bond network, the slower will be the breathing motions which play a role in ligand release.^44^

In this study, we have put forth the atomistic scale mechanism of action of the 4′-DTMP that is effective on a high resistance-conferring mutant of DHFR. This is a proof-of-concept work that shows that focusing on the dynamical mutant-inhibitor interactions through computational means can save time and money for practical designs for strains that need urgent preventive measures due to antibiotic resistance.

## Data Availability

All codes used for the analyses presented here can be found at https://github.com/midstlab/JCIM_Cetin_etal_2023 (those for Figures 2, 4, 6, and 8) and https://github.com/midstlab/JCIM_Cetin_etal_2023_community (those for Figure 7). The trajectories are listed at https://zenodo.org/record/7966540.

## Supporting Information Available

Force field parameters for 4′-DTMP derivatives (Table S1), hydrogen occupancy data and distributions (Table S2 and Figure S5), enzyme-ligand interactions (Figure S1), RMSD, RMSF and cross-correlations from MD simulations (Figures S2–S4, S6) (PDF)
