# Supplementary material for: Kinetic Barrier to Enzyme Inhibition Is Manipulated by Dynamical Local Interactions in E. coli DHFR
Source: J Chem Inf Model. 2023 Jul 26;63(15):4839–49. doi: 10.1021/acs.jcim.3c00818 (PMC10428214; doi:10.1021/acs.jcim.3c00818)
Supplement: Supplementary file 1 — ci3c00818_si_001.pdf [file ci3c00818_si_001.pdf]

Supporting Information for

Kinetic Barrier to Enzyme Inhibition is Manipulated by Dynamical Local Interactions in *E. coli* DHFR

Ebru Cetin[1], Tandac F. Guclu[1], Isik Kantarcioglu[1,2], Ilona K. Gaszek[2], Erdal Toprak[2], Ali Rana Atilgan[1], Burcu Dedeoglu[3*], Canan Atilgan[1*]

[1] Faculty of Engineering and Natural Sciences, Sabancı University, Tuzla, 34956, Istanbul, Turkiye

[2] Department of Pharmacology, University of Texas Southwestern Medical Center, 75390, Dallas, TX, USA

[3] Department of Chemistry, Gebze Technical University, Gebze, 41400, Kocaeli, Turkiye

*Corresponding authors

ebrucetin@sabanciuniv.edu, tguclu@sabanciuniv.edu, kantarcioglu@sabanciuniv.edu, ilona.gaszek@utsouthwestern.edu, erdal.toprak@utsouthwestern.edu, atilgan@sabanciuniv.edu, b.dedeoglu@gtu.edu.tr, canan@sabanciuniv.edu

Table S1. Forcefield parameters of 4'-DTMP in neutral (RESI D4TP) and protonated forms (RESI D4PP).

```
RESI D4TP          0.0
ATOM  NA2 NN1T -0.67800  !        HA41  HA42
ATOM HA21 HN1T  0.30800  !          \  /
ATOM HA22 HN1T  0.30800  !           NA4       H2P       H81
ATOM   C2  CAT  0.61000  !            |         |         |
ATOM   N3 NC2T -0.64500  !           C4   C7    C2P     O3P-C8-H81
ATOM   N1 NC2T -0.71300  !          / \\ /  \  /  \\   /    |
ATOM   C6  CAT  0.41600  !         N3  C5    C1P   C3P    H81
ATOM   H6  HPT  0.09000  !         ||   |    ||    |
ATOM   C4  CAT  0.37100  !  HA21   C2   C6   C6P  C4P
ATOM  NA4 NN1T -0.67300  !   \   / \  //      \  // \
ATOM HA41 HN1T  0.35100  !    NA2   N1         C5P  O4P
ATOM HA42 HN1T  0.35100  !   /                /      \
ATOM   C5  CAT  0.09100  ! HA22             O5P      H4P
ATOM   C7 CT2T -0.36900  !                   |
ATOM  H71  HAT  0.09000  !            H101-C10-H102
ATOM  H72  HAT  0.09000  !                   |
ATOM  C1P  CAT -0.14000  !                  H103
ATOM  C2P  CAT -0.10400  !
ATOM  H2P  HPT  0.09000  !
ATOM  C3P  CAT  0.29100  !
ATOM  O3P  OST -0.45400  !
ATOM   C8 CT3T -0.01300  !
ATOM  H81  HAT  0.09000  !
ATOM  H82  HAT  0.09000  !
ATOM  H83  HAT  0.09000  !
ATOM  C4P  CAT  0.05600  !
ATOM  O4P  OST -0.46100  !
ATOM  H4P  HOM  0.38700  !
ATOM  C5P  CAT  0.29100  !
ATOM  O5P  OST -0.45400  !
ATOM  C10 CT3T -0.01300  !
ATOM H101  HAT  0.09000  !
ATOM H102  HAT  0.09000  !
ATOM H103  HAT  0.09000  !
ATOM  C6P  CAT -0.10400  !
ATOM  H6P  HPT  0.09000  !

BOND NA2 HA21  NA2 HA22  NA2 C2
BOND C2 N1  C2 N3  N3 C4  N1 C6
BOND C6 H6  C6 C5  C4 NA4  C4 C5
BOND NA4 HA41  NA4 HA42  C5 C7  C7 H71
BOND C7 H72  C7 C1P  C1P C2P  C1P C6P
BOND C2P H2P  C2P C3P  C3P O3P  C3P C4P
BOND O3P C8  C8 H81  C8 H82  C8 H83
BOND C4P O4P  C4P C5P  O4P H4P  C5P C6P
BOND C5P O5P  O5P C10  C10 H103  C10 H101
BOND C10 H102  C6P H6P
```

```
RESI  D4PP     1.00
GROUP
ATOM  NA2 NN1T    -0.671000  !         HA41  HA42
ATOM HA21 HN1T     0.499000  !           \   /
ATOM HA22 HN1T     0.499000  !            NA4       H2P       H81
ATOM   C2 CAT     -0.166000  !            |         |         |
ATOM   N3 NC2T    -0.544000  !            C4   C7    C2P     O3P-C8-H81
ATOM   N1 NC2T    -0.643000  !           / \\ /  \  /  \\   /   |
ATOM   C6 CAT      0.501000  !         N3  C5    C1P   C3P    H81
ATOM   H6 HPT      0.090000  !         ||  |    ||    |
ATOM   C4 CAT      0.417000  !  HA21   C2   C6   C6P  C4P
ATOM  NA4 NN1T    -0.622000  !    \   / \  //      \  // \
ATOM HA41 HN1T     0.399000  !    NA2   N1         C5P  O4P
ATOM HA42 HN1T     0.399000  !   /      |         /      \
ATOM   C5 CAT      0.163000  ! HA22      H11     O5P       H4P
ATOM   C7 CT2T    -0.575000  !                   |
ATOM  H71 HAT      0.090000  !            H101-C10-H102
ATOM  H72 HAT      0.090000  !                   |
ATOM  C1P CAT     -0.410000  !                 H103
ATOM  C2P CAT      0.534000  !
ATOM  H2P HPT      0.090000  !
ATOM  C3P CAT      0.290000  !
ATOM  O3P OST     -0.506000  !
ATOM   C8 CT3T    -0.601000  !
ATOM  H81 HAT      0.090000  !
ATOM  H82 HAT      0.090000  !
ATOM  H83 HAT      0.090000  !
ATOM  C4P CAT      0.284000  !
ATOM  O4P OST     -0.629000  !
ATOM  H4P HOM      0.131000  !
ATOM  C5P CAT      0.290000  !
ATOM  O5P OST     -0.506000  !
ATOM  C10 CT3T    -0.601000  !
ATOM H101 HAT      0.090000  !
ATOM H102 HAT      0.090000  !
ATOM H103 HAT      0.090000  !
ATOM  C6P CAT      0.534000  !
ATOM  H6P HPT      0.090000  !
ATOM  H11 HN2      0.544000
BOND NA2 HA21  NA2 C2  NA2 HA22
BOND C2 N1  C2 N3  N3 C4  N1 C6
BOND N1 H11  C6 H6  C6 C5  C4 NA4
BOND C4 C5  NA4 HA41  NA4 HA42  C5 C7
BOND C7 H71  C7 C1P  C7 H72  C1P C2P
BOND C1P C6P  C2P H2P  C2P C3P  C3P C4P
BOND C3P O3P  O3P C8  C8 H82  C8 H83
BOND C8 H81  C4P C5P  C4P O4P  O4P H4P
BOND C5P C6P  C5P O5P  O5P C10  C10 H101
BOND C10 H103  C10 H102  C6P H6P
```

Selection of hydrogen bonds whose occupancies display significant deviation from WT^{TMP}

The 1 μs trajectory is split into five chunks. Bonds that show 20 % (3σ; see Figure S5) change from the average occupancy having less than 2σ deviation are chosen for further refinement. The bonds that pass average criteria but fails standard deviation are shown in dark gray. The bonds that fail both criteria are shown in light grey. The bonds that hold both criteria are shown in bold black and boxed.

Table S2a. WT^{TMP} hydrogen bond occupancy changes with respect to WT^{TMP}.

		Chunk 1	Chunk 2	Chunk 3	Chunk 4	Chunk 5	% deviation from WT
$L28R^{TMP}$	**ASP37-ARG57**	**-0.12**	**-0.22**	**38.24**	**60.12**	**67.01**	**33±29**
	ARG159-ARG158	-1.12	-1.12	27.15	48.63	7.27	16±19
	ASP69-TRP47	2.59	0.4	14.98	11.29	24.37	11±9
	ARG158-ARG159	10.11	-0.18	17.9	23.1	1.92	11±9
	SER148-ASP144	-0.68	3.12	20.9	17.3	7.01	10±8
	THR46-SER49	1.98	22.16	9.47	3.98	-0.22	7±8
	ALA19-MET16	36.73	7.16	-6.53	-6.83	-7.13	5±17
	LYS38-ARG57	-0.76	-0.76	-0.76	-0.76	24.51	4±10
	ASP127-ARG12	31.39	-7.57	7.31	-6.97	-4.78	4±15
	ASP132-ARG159	1.83	-23.34	6.03	11.82	9.03	1±13
	ASP69-ARG71	-5.27	2.22	12.81	11.91	-32.64	-2±17
	ASN59-LEU54	0.12	-7.27	-14.26	-9.67	-23.46	-11±8
	GLU134-ARG159	-13.83	-38.8	-7.74	0.36	1.26	-12±15
	GLU90-LYS109	-3.88	-15.17	-15.97	-23.26	-4.08	-12±7
	LYS58-VAL40	0.46	-7.24	-15.93	-29.51	-12.13	-13±10
	GLU101-ARG98	-0.66	-1.76	-20.74	-33.73	-16.85	-15±12
	GLU157-GLU134	-20.32	-34.71	-14.73	-2.44	-3.84	-15±12
	ASP27-PHE31	-9.47	-20.16	-19.96	-11.37	-17.26	-16±4
	ASN147-ASP144	-0.32	-9.22	-24.6	-24.6	-24.6	-17±10
	ASP132-ARG158	-5.64	10.65	-28.01	-48.69	-17.82	-18±20
	PRO55-ARG57	-9.91	-9.91	-12.31	-30.69	-30.69	-19±10
	**THR35-ARG57**	**-16.74**	**-13.84**	**-22.23**	**-25.23**	**-25.23**	**-21±5**
	**THR46-ILE50**	**-12.7**	**-25.29**	**-22.89**	**-23.09**	**-20.7**	**-21±4**
	**GLU129-ARG158**	**-2.98**	**12.2**	**-35.45**	**-65.02**	**-25.86**	**-23±27**
	**ASP144-ASN147**	**-14.15**	**-22.74**	**-42.42**	**-42.42**	**-42.42**	**-33±12**

Table S2b. WT$^{4'-DTMP}$ hydrogen bond occupancy changes with respect to WT^{TMP}.

		Chunk 1	Chunk 2	Chunk 3	Chunk 4	Chunk 5	% deviation from WT
WT$^{4'-DTMP}$	**ALA19-MET16**	**18.84**	**46.42**	**38.62**	**44.52**	**39.62**	**38±10**
	**ASP144-SER148**	**-1.18**	**11.71**	**10.51**	**47.67**	**30.89**	**20±17**
	ARG159-ARG158	31.15	23.95	11.77	5.77	7.87	16±10
	ASP144-GLN146	-5.4	-9.1	0.5	21.57	18.58	5±13
	ASP144-TYR151	1.56	21.54	0.16	-1.64	-1.64	4±9
	GLU101-ARG98	-25.24	-12.75	-6.36	3.73	-2.66	-9±10
	LEU8-ILE115	-17.6	-24.1	-2.82	-2.02	-3.32	-10±9
	ASP144-ASN147	-19.84	-35.33	-8.35	-8.95	-6.76	-16±11
	ASN147-ASP144	-8.62	-22.7	-14.21	-24.6	-24.6	-19±6
	GLU48-ARG44	5.77	-17.6	-24.6	-27.19	-32.49	-19±13
	**GLU90-TYR111**	**-18.16**	**-14.27**	**-37.24**	**-32.75**	**-23.66**	**-25±9**
	**GLU90-LYS109**	**-29.65**	**-14.67**	**-33.05**	**-29.95**	**-22.76**	**-26±7**
	**ASP132-ARG158**	**-44.2**	**-34.11**	**-19.22**	**-14.83**	**-20.72**	**-27±11**
	**GLU129-ARG158**	**-57.53**	**-39.85**	**-32.15**	**-22.96**	**-26.66**	**-36±12**

Table S2c. L28R $^{4'-DTMP}$ hydrogen bond occupancy changes with respect to WT^{TMP}.

		Chunk 1	Chunk 2	Chunk 3	Chunk 4	Chunk 5	% deviation from WT
L28R $^{4'-DTMP}$	**ALA19-MET16**	**10.45**	**31.93**	**38.42**	**44.32**	**45.92**	**34±13**
	**ARG159-ARG158**	**16.16**	**2.88**	**3.78**	**40.14**	**45.13**	**22±18**
	GLU154-SER135	20.95	9.97	23.25	21.35	18.96	19±5
	ASP142-ASN147	0.38	0.68	3.68	36.04	23.56	13±14
	ARG158-ARG159	6.61	6.01	6.71	18.3	23.99	12±7
	PRO126-ARG12	4.6	0.5	-0.1	0	29.67	7±12
	GLU139-ARG33	0.8	0.6	29.07	0.2	1	6±11
	GLU157-ARG159	-4.47	3.22	-5.87	14.21	21.3	6±11
	ASP79-SER77	-4.83	-7.93	26.03	-4.44	-2.34	1±12
	SER150-ASP116	-5.27	-27.65	-1.88	-3.48	-3.08	-8±10
	GLU90-TYR111	-5.57	-13.57	-9.17	-21.56	-16.16	-13±6
	LEU8-ILE115	-13.41	-41.08	-17.4	-0.22	0.48	-14±15
	VAL13-ALA9	-12.62	-16.32	-24.91	-15.32	-9.82	-16±5
	ASN147-ASP144	-4.52	-12.81	-21.2	-24.6	-21.4	-17±7
	GLU90-LYS109	-11.77	-12.77	-12.77	-25.26	-26.35	-18±7
	THR35-ARG57	-17.44	-17.04	-19.14	-20.14	-19.44	-19±1
	GLU101-ARG98	-31.63	-10.75	-23.14	-9.66	-21.14	-19±8
	**ASP127-ARG12**	**-17.46**	**-22.16**	**-14.67**	**-22.66**	**-29.55**	**-21±5**
	**GLU48-ARG44**	**-10.01**	**-28.09**	**-34.59**	**-31.09**	**-34.49**	**-28±9**
	**ASP132-ARG158**	**-27.01**	**-14.93**	**-29.81**	**-43.7**	**-47.39**	**-33±12**
	**ASP144-ASN147**	**-15.35**	**-34.33**	**-38.92**	**-42.02**	**-39.02**	**-34±10**
	**GLU129-ARG158**	**-39.45**	**-21.26**	**-33.25**	**-52.53**	**-65.02**	**-42±15**

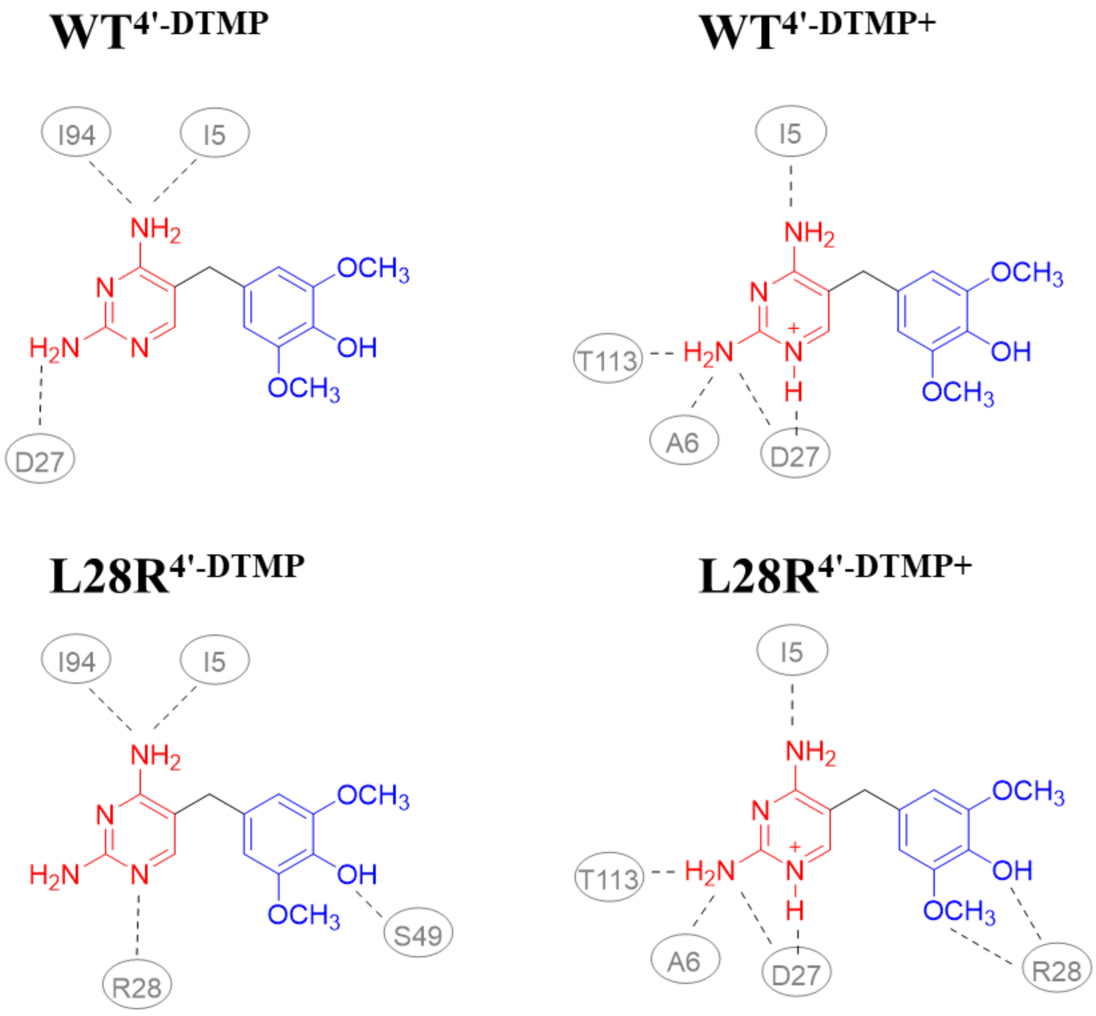

Figure S1. Enzyme-ligand interactions for WT and L28R with 4'-DTMP. The interactions that appear more than %5 are shown for the 210 ns-long simulations.

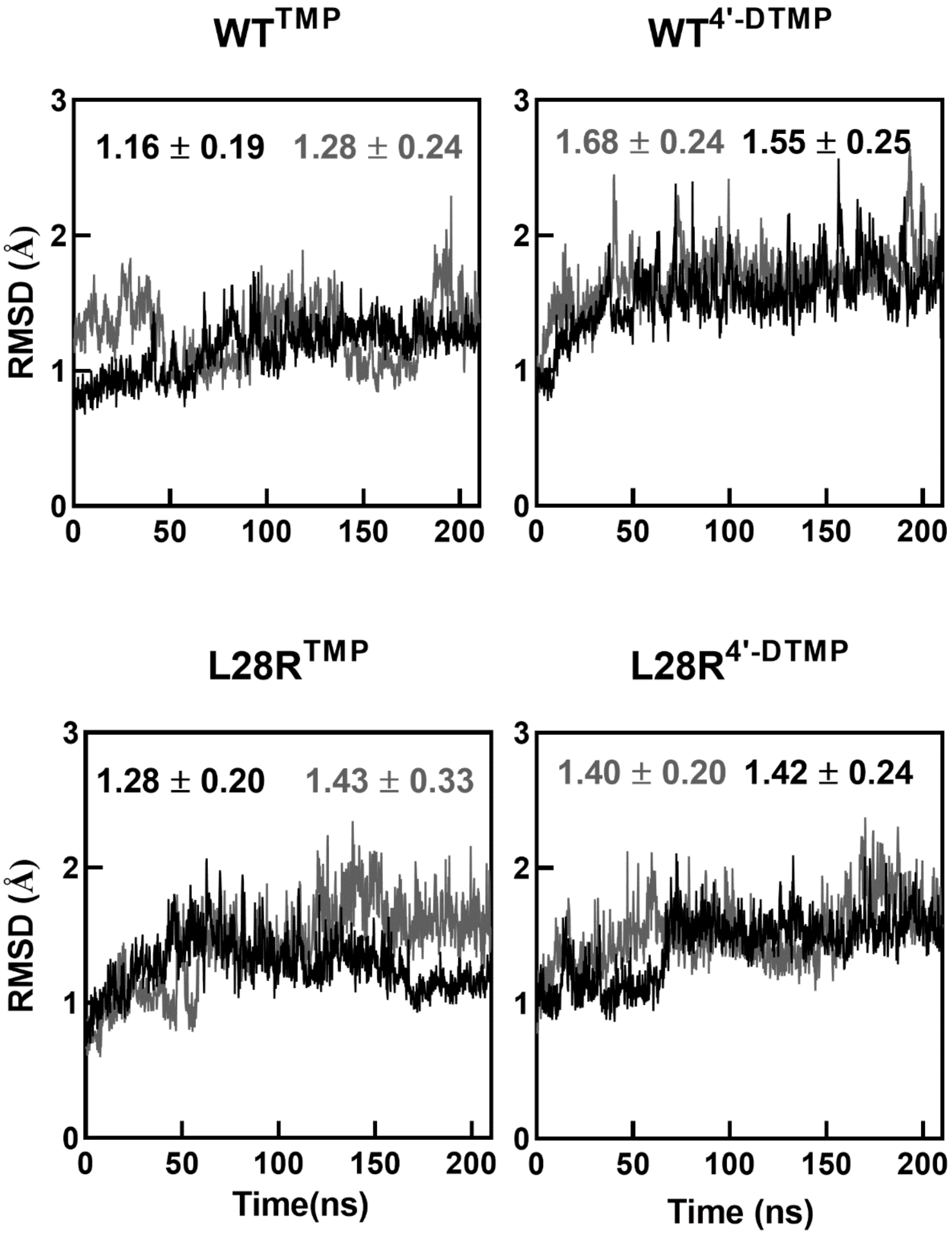

Figure S2. RMSD profiles of WT and L28R with TMP and 4'-DTMP bound for the two replicates (black and gray) of 210ns-long simulations. The corresponding average RMSDs are reported inside the plots.

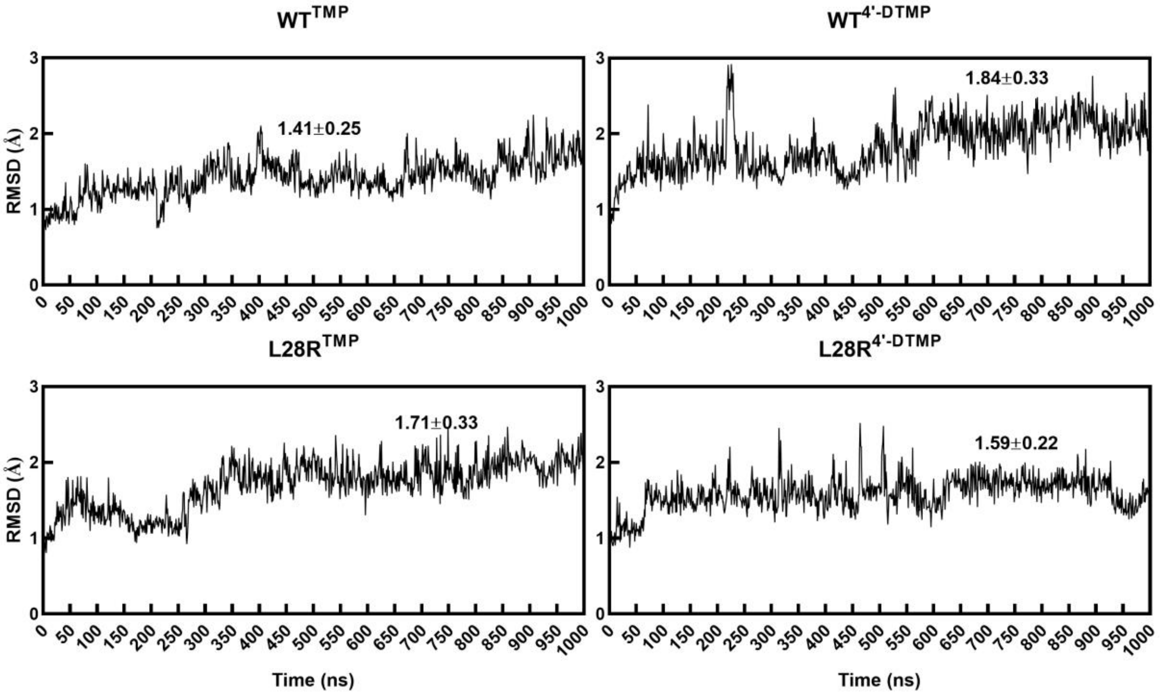

Figure S3. RMSD profiles for 1 μs-long trajectories. The corresponding average RMSDs are reported inside the plots.

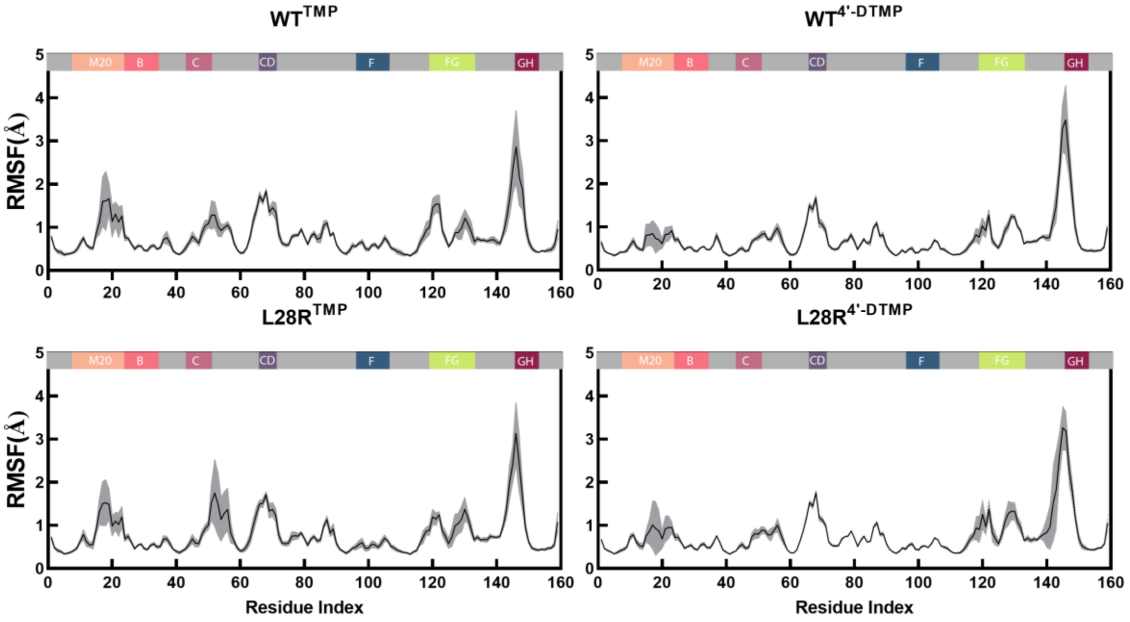

Figure S4. The mean squared fluctuations of the systems studied. These are calculated over 200ns chunks of all trajectories; standard deviations are shown in the shaded regions. When 4'-DTMP is bound the loops contacting the ligand loses flexibility and becomes more stable in both the WT and the mutant.

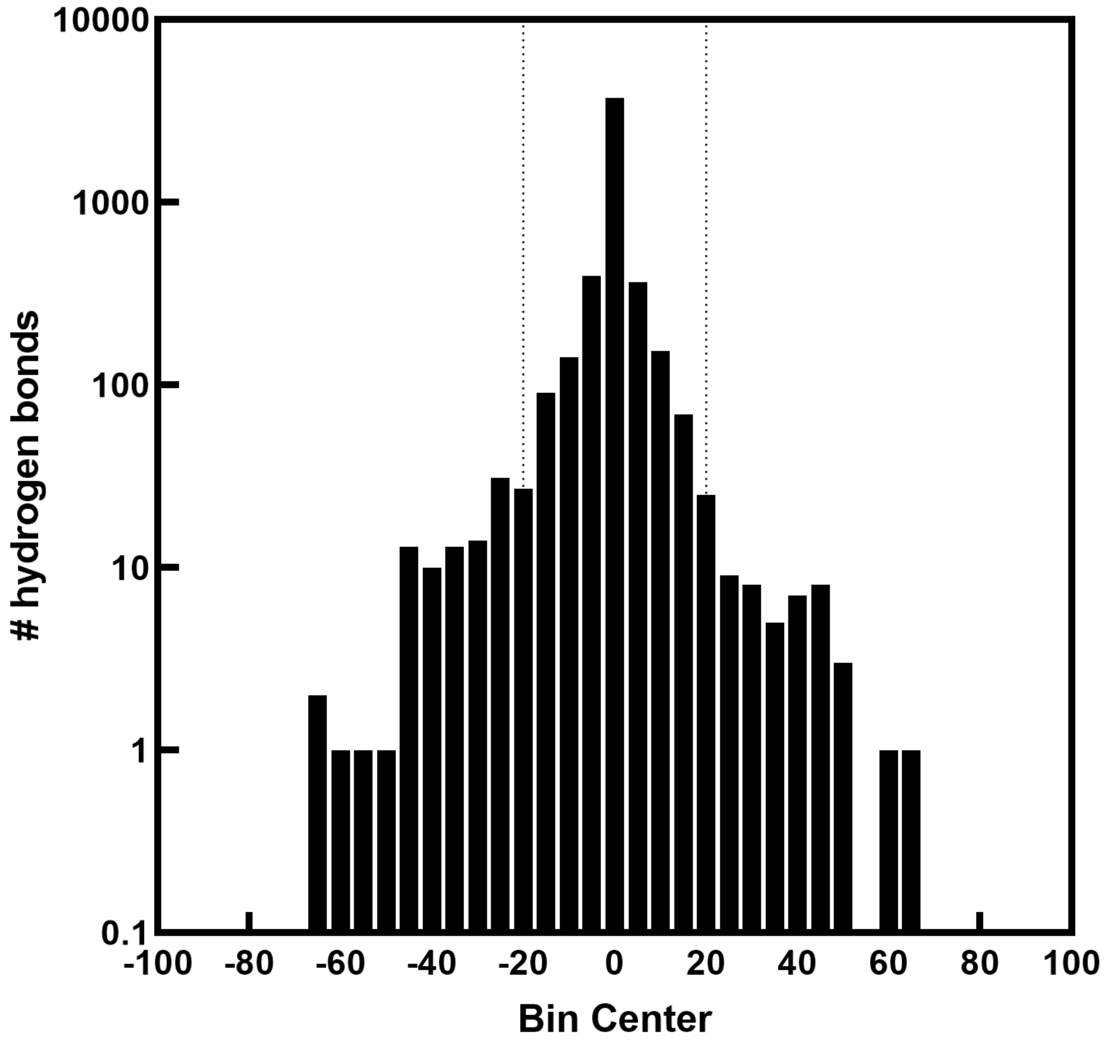

Figure S5. Semi-log cumulative frequency distribution of the change in hydrogen bonding occupancies with respect to the WT^{TMP} system. A bin size 5 % is used. Vertical dashed lines indicate three standard (σ = 6.6%) deviations of the mean which correspond to 20 %.

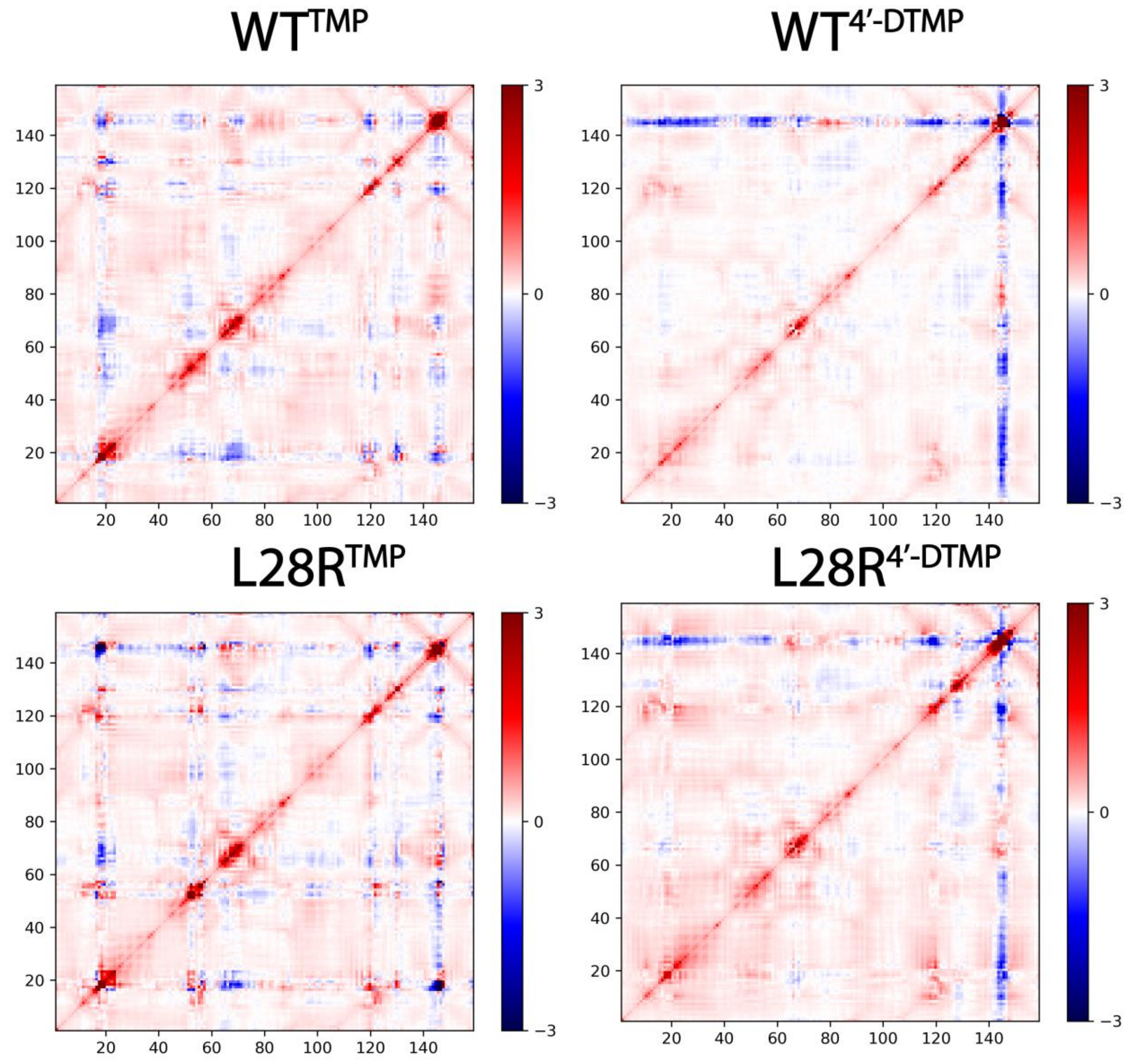

Figure S6. Cross-correlation maps of WT, L28R TMP-bound trajectories and its derivatives. Cross-correlations are calculated over C_β atoms (C_α for glycine). For ligands, ring carbon and the oxygen of the trimethoxy group are selected as nodes.
